# A reduced concentration of brain interstitial amino acids is associated with depression in subarachnoid hemorrhage patients

**DOI:** 10.1038/s41598-019-39569-5

**Published:** 2019-02-26

**Authors:** Mario Kofler, Alois Josef Schiefecker, Maxime Gaasch, Barbara Sperner-Unterweger, Dietmar Fuchs, Ronny Beer, Boris Ferger, Verena Rass, Werner Hackl, Paul Rhomberg, Bettina Pfausler, Claudius Thomé, Erich Schmutzhard, Raimund Helbok

**Affiliations:** 1 Neurological Intensive Care Unit, Department of Neurology, Medical University of Innsbruck, Innsbruck, Austria; 2 Department of Psychiatry, Psychotherapy and Psychosomatics, University Hospital of Psychiatry II, Medical University of Innsbruck, Innsbruck, Austria; 3 Division of Biological Chemistry, Biocenter, Medical University of Innsbruck, Innsbruck, Austria; 4 CNS Diseases Research, Boehringer Ingelheim Pharma GmbH & Co. KG, Biberach an der Riss, Germany; 5 Medical Informatics and Technology, UMIT – University for Health Sciences, Hall, Austria; 6 Department of Neuroradiology, Medical University of Innsbruck, Innsbruck, Austria; 7 Department of Neurosurgery, Medical University of Innsbruck, Innsbruck, Austria

## Abstract

The amino-acids tryptophan, phenylalanine and tyrosine seem to play an important role in the pathophysiology of depressive disorders. We measured daily brain extracellular levels of these amino-acids using cerebral microdialysis (CMD) and high performance liquid chromatography in 26 consecutive subarachnoid hemorrhage (SAH) patients and associated them with the presence of depressive disorders. Patients were grouped as follows: medical history of depression (prior to SAH), antidepressant intake 12 months after SAH (but not before), or neither. CMD-tryptophan, CMD-phenylalanine and CMD-tyrosine levels were significantly lower in patients with preexisting depressive disorders compared to those without depression (p < 0.01). Disease severity and SAH-related complications were not associated with amino-acid concentrations. We found a positive correlation between nutritionally administered and brain interstitial levels of tryptophan and phenylalanine in non-depressed patients (R = 0.26 and R = 0.24, p < 0.05), which was not present in patients with preexisting depression (p > 0.1). In conclusion, brain interstitial levels of tryptophan, phenylalanine and tyrosine measured in the context of the clinical management of SAH were significantly decreased in patients with a medical history of depression. This study supports the hypothesis that the availability of these neurotransmitter precursor amino-acids in the human brain may play an important role in the pathophysiology of depressive disorders.

## Introduction

Despite the increasing number of survivors, aneurysmal subarachnoid hemorrhage (SAH) remains a devastating disease with high morbidity and poor recovery^1–3^. Up to half of the survivors do not return to their previous level of employment, which further aggravates the tremendous socioeconomic burden caused by SAH^3,4^. In addition to traditional functional outcome measures, recent research has focused on neuropsychological outcome and mood disturbances as complementary parameters. Symptoms of depression occur in up to 50% of patients after SAH^3^, even in those living independently^5^, and the use of antidepressant medication is much more common compared to matched controls^6^. Depression after SAH is associated with reduced quality of life^7^, higher risk of not returning to previous work^8^, and is one of the major causes of poor recovery^9^.

Pathophysiologic mechanisms underlying depressive disorders are still incompletely understood. The “monoamine-deficiency hypothesis” claims that a decrease of the neurotransmitters serotonin and noradrenalin in the brain contributes to the development of depressive disorders^10^. Both neurotransmitters are synthesized from aromatic amino-acids, i.e. serotonin from tryptophan, and noradrenalin from tyrosine or its precursor phenylalanine. A shortage or impaired processing of these amino-acids may therefore result in decreased serotonin and noradrenalin levels.

Systemic chronic inflammatory processes are the most extensively discussed mechanism interfering with the synthesis of serotonin and noradrenalin from their respective precursor amino-acids^11^. Pro-inflammatory cytokines induce the enzyme indoleamine 2,3-dioxygenase, which leads to an alternative pathway of tryptophan breakdown, converting it to kynurenine and potentially neurotoxic downstream products^12^. Prolonged inflammation and formation of reactive oxygen species may lead to a depletion of antioxidant systems including tetrahydrobiopterin (BH_4_). BH_4_ plays a key role in the synthesis of both, serotonin and noradrenalin. In the noradrenergic pathway, BH_4_ is needed for both, the conversion of phenylalanine to tyrosine and the further processing to levodopa (L-3,4-dihydroxyphenylalanine). In line with this pathophysiologic concept, decreased levels of tryptophan and increased levels of phenylalanine and tyrosine, as well as an increased phenylalanine-to-tyrosine ratio, were found in serum of patients with depressive disorders^13^. So far it is unknown if these changes also occur in the brain and if such profiles can be measured in human brain tissue.

Cerebral microdialysis (CMD) allows the quantification of small molecules in the cerebral extracellular space in patients suffering from acute brain injury. The feasibility of measuring brain interstitial levels of tryptophan, phenylalanine and tyrosine in SAH patients has already been demonstrated^14,15^, however, an association of cerebral concentrations of these amino-acids with depression has not been investigated so far.

The aim of this study was to associate levels of tryptophan, phenylalanine and tyrosine, assessed directly in human brain tissue by CMD, with preexisting depressive disorders in SAH patients and newly occurred depressive disorders following SAH.

## Materials and Methods

### Patient selection, data collection and ethical approval

Data analyzed in this study were collected in two ongoing prospective observational registry studies aiming to include (1) all patients with non-traumatic SAH and (2) all patients undergoing invasive multimodal neuromonitoring (as part of routine clinical management in comatose neurocritical care patients) admitted to the neurological intensive care unit at the Medical University of Innsbruck, Austria. Both studies were approved by the ethics committee of the Medical University of Innsbruck (AN3898 285/4.8, AM4091-292/4.6, UN3898 285/4.8). In our department CMD, together with other established invasive probes, is only performed in mechanically ventilated SAH patients (either on admission or after secondary neurologic deterioration) for attempting early detection of impending secondary brain injury, as medically indicated. Thus the necessity of initiating invasive neuromonitoring is unforeseeable and informed consent cannot be obtained a priori. Deferred written informed consent for the collection of data was obtained from all patients according to Austrian law as soon as the individual regained the ability to consent (awake and fully oriented patient and after the cessation of sedative medication) by a qualified member of the study team or from a legally authorized representative, as soon as one was available. The concept of proxy consent is not supported by Austrian law, however the relatives or next of kin, respectively, were informed about invasive neuromonitoring procedures. The inclusion criteria for this study were (1) admission with non-traumatic SAH, (2) ≥18 years of age, and (3) invasive neuromonitoring including CMD catheter placement. The CMD samples used were collected between 2010 and 2012 from all 26 consecutive SAH patients undergoing CMD-monitoring included in our institutional neuromonitoring registry. Sample size calculation was not possible due to lack of data investigating the same hypothesis. We predefined a sample size of at least 25 patients. Amino acid quantification was performed at one point after all patients had been included and no statistical analysis was performed before all values were available.

The patient’s baseline characteristics, interventions, hospital complications and functional outcome were recorded in our institutional SAH registry. Intracranial pressure (ICP) and cerebral perfusion pressure (CPP, mean arterial pressure measured at the foramen of Monro minus ICP) were saved as 3-minute average in our patient data management system (Centricity^TM^ Critical Care 7.0 SP2; GE Healthcare Information Technologies, Dornstadt, Germany) and aggregated over the microdialysis sampling time (approximately one hour). ICP was measured by a parenchymal probe (NEUROVENT-P-TEMP; Raumedic®, Helmbrechts, Germany). The CMD-probe (71 High Cut-Off Brain Microdialysis Catheter, membrane length 1 cm, pore size 100 kDa; M Dialysis AB, Stockholm, Sweden) was tunneled and inserted into the white matter of the watershed of anterior and middle cerebral artery ipsilateral to the aneurysm or the vascular territory being deemed to be at greatest risk of developing secondary brain injury. Isotonic perfusion fluid (Perfusion Fluid CNS; M Dialysis AB, Stockholm, Sweden) was pumped through the system at a flow rate of 0.3 μl/min. Bedside analysis of CMD samples for glucose, pyruvate, lactate and glutamate concentrations for clinical routine care was performed using the CMA 600 and Iscus^flex^ (M Dialysis AB, Stockholm, Sweden). The first sample was taken at least one hour after probe insertion and discarded to avoid placement-related artifacts. After bedside analysis CMD samples were stored at −80 °C. Metabolic distress was defined as a lactate-to-pyruvate-ratio (LPR) >40, mitochondrial dysfunction as LPR >30 together with CMD-pyruvate >70 µmol/l and neuroglucopenia as CMD-glucose <0.7 mmol/l.

### Measurement of amino-acid concentrations

For the daily assessment of amino acid concentrations, the CMD sample at 6:00 am (time of routine blood sampling) was taken. Amino-acid levels were measured by high-performance liquid chromatography (HPLC) using fluorescence detection and ortho-phthalaldehyde/2-mercaptoethanol (OPA/MCE) derivatization, recently modified to measure *in vivo* microdialysates. The HPLC system consisted of an HPLC pump (P680), an automated sample injector (ASI-100T), and a fluorescence detector (RF 2000; all Dionex, Idstein, Germany). Fifteen µl of the sample was mixed with 3 µl OPA/MCE derivatization reagent incubated for 3 min before injection onto the analytical column (Nucleosil 120 C18, 5 µm, 60.0 × 4.0 mm) attached to a precolumn (Nucleosil 120 C18, 5 µm, 5.0 × 4.0 mm, both MZ Analysentechnik, Mainz, Germany) maintained at a stable temperature of 30 °C using a column oven. Chromatographic separation was achieved within 25 min using the following gradient at a constant flow rate of 1.2 ml/min. Mobile phase A (0.1 M sodium acetate buffer containing 10% methanol, pH 6.95,) and mobile phase B (97,5% methanol/2,5% tetrahydrofuran) were used for the following gradient: 0.0–6.0 min 100% A; 8.0–1.0 min 90% A; 15.0 min 75% A; 18.0–21.0 min 60% A; 23.0 min 40% A; 25.5 min 15% A; 25.8–27.5 min 0% A; 28.5–30.0 min 10% A. The fluorescence detector operated at an excitation wavelength of 345 nm and emission wavelength of 450 nm. In case of samples with amino-acid levels outside of the linear range, the samples were diluted (1:100). The HPLC software Chromeleon™ 6.60 (Dionex, Idstein, Germany) served for data acquisition and analysis. For method validation peak identity of all measured amino acids was confirmed by spiking experiments in the microdialysate matrix and evaluation of the peak shape and the retention time.

### Grading and radiologic definitions

The clinical severity of the SAH was graded using the Hunt & Hess (H&H) scale and overall morbidity using the Acute Physiology and Chronic Health Evaluation II (APACHE-II) score. Computed tomography (CT) was performed on admission, after aneurysm treatment and when clinically indicated. Images were graded by a neuro-radiologist blinded to clinical parameters and amino acid concentrations using the modified Fisher-score, the SAH sum-score, the intraventricular hemorrhage sum-score and screened for the presence of global cerebral edema^16–18^. CMD probe location was defined as “perilesional” if the gold tip of the probe was within 1 cm to a focal hypodense (edema/infarction) or hyperdense lesion (hematoma) on brain CT imaging, or, if there was no focal brain lesion within 1 cm of the tip of the probe, as “normal-appearing brain tissue”.

### Patient care

The clinical care of our SAH patients is based on current international recommendations^19,20^. Ruptured aneurysms were diagnosed either by CT angiography or conventional angiography and treated with clipping or coiling. An external ventricular drain (EVD) was placed in case of hydrocephalus. Intravenous fluids (primarily crystalloids) and vasopressors (primarily noradrenalin) were used to achieve an adequate cerebral perfusion pressure (above 70 mmHg, unless indicated otherwise). All patients were comatose during the period of invasive neuromonitoring and routinely received continuous intravenous sedation. All patients received prophylactic intravenous nimodipine and were screened for vasospasm with transcranial color-coded duplex sonography. Delayed cerebral ischemia (DCI) was defined as a new focal neurological impairment, a decrease of at least two points on the Glasgow Coma Scale, or a new infarct on CT or MRI, not attributable to other causes^21^. The modified Rankin Scale (mRS) was used to assess functional outcome three months after the bleeding. An mRS score of smaller than four was defined as good functional outcome.

### Depression

Patients were categorized into four groups: (1) patients in whom the diagnosis of a depressive disorder was made or at least confirmed by a psychiatrist (screening of all available medical documents/charts) at any time before the bleeding and who took antidepressant medication when SAH occurred (DEP_prior+_); (2) patients who took antidepressant medication at their 12-month follow-up visit after the SAH, but not on admission, and did not have a medical history of depression (screening of all available medical documents/charts) (DEP_post+_); (3) patients without a medical record of a depressive disorder prior to the bleeding and no antidepressant medication on admission and 12 months after SAH (screening of all available medical documents/charts, anamnesis at 12-month follow-up) (DEP_no_); (4) patients who died within 12 months after SAH (none of whom had a medical history of a depressive disorder or was on antidepressant medication on admission).

### Nutrition

Our institutional nutrition protocol for mechanically ventilated patients aims at providing 20–25 kilocalories per kilogram bodyweight per day. Enteral nutrition is the preferred way of feeding. It is initiated within 24 hours after aneurysm treatment and increased over the first 3 days. During this time, parenteral amino acid solution is administered in addition. If the caloric goal cannot be achieved by day 4, supplemental parenteral nutrition (all-in-one emulsion of glucose, amino acids and lipids) is considered. The standard products used in these patients were Isosource^®^ Standard Neutral SmartFlex^®^ (Nestlé Austria, Vienna), Aminoven^®^ 3,5% GE and StructoKabiven^®^ (both Fresenius Kabi Austria, Graz), all of which contain phenylalanine, tyrosine and tryptophan.

### Statistics

Continuous variables are reported as mean ± standard error of mean (SEM) or median and interquartile range (IQR) as appropriate. Categorical variables are reported as count and percentage in each group. Time-series data were analyzed using generalized linear models with a normal distribution and identity-link function, which were extended by generalized estimating equations (GEE) with an autoregressive process of the first order to account for repeated measurements within individual patients. Before being added to the GEE model, non-normally distributed continuous variables underwent Log-transformation to meet assumptions of normality. To assess temporal dynamics of amino-acid concentrations, days were pooled in three groups (days 0–3, 4–7 and 8–10). These groups were compared in a GEE model with amino-acid levels as dependent and the group of days as independent variable. Similarly, the association between depression groups and cerebral amino-acid levels and the association between depression groups and other neuromonitoring data was assessed. Spearman’s rho was used to analyze the correlations between cerebral and nutritional amino-acids. All analyses were performed with IBM-SPSS V22.0 (SPSS Inc., Chicago, IL, USA). The threshold for statistical significance was set at a *P*-value < 0.05.

## Results

Baseline characteristics, disease severity, radiologic grading, hospital complications and functional outcome of 26 consecutive SAH-patients with CMD are summarized in Table 1. Neuromonitoring was initiated on day 1 (1–2) after SAH. Probe location was classified as normal-appearing brain tissue in 12 (46%) and as perilesional in 14 patients (54%).

**Table 1:** Patient Characteristics, disease severity, complications and functional outcome.

Characteristics, complications, outcome	n (%) or median (IQR)
Age (years)	55 (47–68)
Gender (female)	15 (58)
Hunt and Hess grade (admission)	
2	2 (8)
3	6 (23)
4	3 (11)
5	15 (58)
Loss of conciousness (initial)	14 (54)
APACHE II score	18 (15–21)
Modified Fisher grade	
1	3 (11.5)
2	3 (11.5)
3	7 (27)
4	13 (50)
SAH sum score	23 (15–27)
IVH sum score	5 (0–10)
Aneurysm size >10 mm	7 (27)
Global cerebral edema	14 (54)
SAH-related parenchymal hematoma	14 (54)
Hydrocephalus requiring EVD	23 (88)
Clipping	19 (73)
Hemicraniectomy	8 (31)
Pneumonia	19 (73)
Sepsis	5 (19)
Delayed cerebral ischaemia	6 (23)
Anemia (requiring transfusion)	14 (54)
Length of ICU stay (days)	38 (25–56)
Modified Rankin scale (after 3 months)	
0	2 (8)
1	3 (12)
2	3 (12)
3	2 (8)
4	4 (15)
5	8 (30)
6	4 (15)

Data of 26 patients are shown as n (%) or median (interquartile range). APACHE II = acute physiology and chronic health evaluation II; SAH = subarachnoid hemorrhage; IVH = intraventricular hemorrhage; EVD = external ventricular drain; ICU = intensive care unit.

### Amino-acid levels over time and associated factors

Absolute mean concentrations of CMD-tryptophan, CMD-phenylalanine and CMD-tyrosine over time are shown in Table 2. The levels of all three amino-acids significantly increased over time (p < 0.01). Except for patient age, none of the other factors (gender, Hunt & Hess grade, APACHE-II score, modified-Fisher score, the presence of GCE, DCI, hydrocephalus, CMD probe location, clipping/coiling) was associated with amino-acid levels (Table 3).

**Table 2:** Absolute brain tissue concentrations of tryptophan, phenylalanine and tyrosine of all patients and groups over time.

	Days 0–3	Days 4–7	Days 8–10	p-value of increase over time
**All patients (n = 26**, **100%)**				
CMD-tryptophan (µM/l)	7.79 ± 0.79	9.2 ± 0.81	10.58 ± 1.05	<0.001
CMD-phenylalanine (µM/l)	34.04 ± 3.3	41.14 ± 3.26	56.66 ± 4.76	<0.001
CMD-tyrosine (µM/l)	22.16 ± 2.35	30.66 ± 2.38	37.48 ± 3.28	<0.001
**DEP**_**no**_ **(n = 11**, **42%)**				
	Days 0–3	Days 4–7	Days 8–10	p-value of increase over time
CMD-tryptophan (µM/l)	10.62 ± 1.51	13.04 ± 16.68	14.52 ± 2.02	<0.001
CMD-phenylalanine (µM/l)	39.95 ± 5.44	55.17 ± 6.03	65.85 ± 8.01	<0.001
CMD-tyrosine (µM/l)	27.63 ± 3.97	41.25 ± 4.61	44.12 ± 6.08	<0.001
**DEP**_**prior+**_ **(n = 4**, **15%)**				
	Days 0–3	Days 4–7	Days 8–10	p-value of increase over time
CMD-tryptophan (µM/l)	2.54 ± 0.19	4.25 ± 0.37	4.62 ± 0.46	<0.001
CMD-phenylalanine (µM/l)	12.23 ± 0.55	20.43 ± 1.99	28.51 ± 2.42	<0.001
CMD-tyrosine (µM/l)	7.24 ± 0.45	15.59 ± 1.57	17.73 ± 1.32	<0.001
**DEP**_**post+**_ **(n = 7**, **27%)**				
	Days 0–3	Days 4–7	Days 8–10	p-value of increase over time
CMD-tryptophan (µM/l)	5.10 ± 0.74	5.83 ± 0.43	7.62 ± 0.74	0.026
CMD-phenylalanine (µM/l)	27.98 ± 5.15	26.65 ± 3.07	51.61 ± 6.48	0.028
CMD-tyrosine (µM/l)	17.89 ± 4.20	21.29 ± 24.40	34.19 ± 4.28	<0.001

Data are shown as mean (standard error of mean). Data were pooled for days 0–3, 4–7 and 8–10 after the subarachnoid hemorrhage. The p-value refers to the difference between the pooled data of days 0–3 and days 8–10. Statistical analysis was performed using a linear, univariate model in generalized estimating equations with amino-acid levels as dependent and the group of days as independent variable. DEP_no_ = patients with neither a medical history of depression nor antidepressant medication use 12 months after the subarachnoid hemorrhage; DEP_prior+_ = patients with a medical history of depression; DEP_post+_ = patients with no medical history of depression but with anti-depressant medication use 12 months after the subarachnoid hemorrhage; CMD = cerebral microdialysis.

**Table 3:** Associations of patient characteristics, disease severity and complications with brain tissue concentrations of tryptophan, phenylalanine and tyrosine.

Factor	CMD-Tyrosine			CMD-Tryptophan			CMD-Phenylalanine		
	OR	95% CI	p-value	OR	95% CI	p-value	OR	95% CI	p-value
Age above 55 years (median)	0.84	0.7–1.004	0.055	0.85	0.72–0.999	0.049*	0.82	0.69–0.98	0.024*
Gender (female)	0.90	0.74–1.090	0.28	0.93	0.78–1.113	0.43	0.89	0.74–1.067	0.20
Poor clinical grade (H&H 4–5)	1,12	0.92–1.38	0.27	1,10	0.91–1.32	0.32	1,07	0.88–1.31	0.49
Poor modified Fisher grade (3–4)	0.90	0.73.1.109	0.32	0.90	0.74–1.097	0.30	0.92	0.75–1.12	0.40
APACHE II score above 18 (median)	0.95	0.77–1.181	0.67	0.99	0.81–1.207	0.91	0.98	0.80–1.12	0.83
Global cerebral edema	1,10	0.90–1.34	0.37	1,09	0.91–1.31	0.35	1,11	0.92–1.34	0.28
Delayed cerebral ischaemia	1,12	0.90–1.41	0.32	1,11	0.89–1.40	0.35	1,14	0.91–1.42	0.24
Hydrocephalus requiring EVD	1,04	0.86–1.26	0.70	1,06	0.87–1.28	0.59	1,10	0.85–1.39	0.50
Probe location (normal-appearing)	0.92	0.79–1.07	0.27	0.92	0.80–1.05	0.21	0.92	0.80–1.06	0.26
Aneurysm clipping (versus coiling)	1,05	0.83–1.33	0.67	1,03	0.82–1.29	0.80	1,05	0.83–1.33	0.67

An asterisk (*) signifies significant associations, which were only found between lower phenylalanine and tyrosine levels and patients older than the median age of 55 years. Statistical analysis was performed using a linear, univariate model in generalized estimating equations with amino-acid levels as dependent and the dichotomized factor as independent variable. CMD = cerebral microdialysis; OR = odds ratio; 95% CI = 95% confidence interval; H&H = Hunt & Hess grade; APACHE-II = acute physiology and chronic health evaluation II; EVD = external ventricular drain.

### Patients with depression before and after SAH

Four patients (15%) had a medical history of depression (DEP_prior+_ patients), of whom 3 suffered from major depressive disorders and were treated with (1) sertraline, (2) mirtazapine and (3) citalopram + trazodone. One patient had a bipolar affective disorder and was treated with lithium. Seven patients (27%) received antidepressant treatment within 12 months after SAH (DEP_post+_ patients; 3 took citalopram, 4 took venlafaxine). Four patients (15%) died, leaving 11 patients (42%) without medical history of depression and no intake of antidepressants 12 months after SAH (DEP_no_ patients).

**Figure 1:**
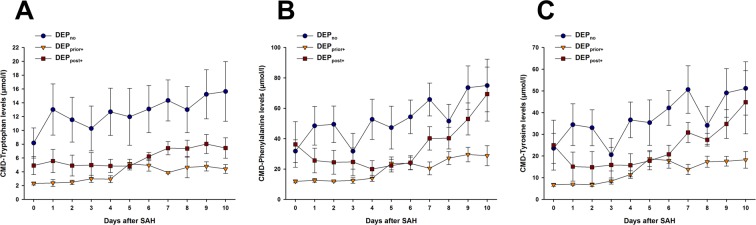
Temporal course of CMD-tryptophan (**A**), CMD-phenylalanine (**B**) and CMD-tyrosine (**C**); patients with a medical history of depression showed significantly lower levels of all 3 amino-acids compared to patients without depression. Patients with antidepressant intake 12 months after subarachnoid hemorrhage showed significantly lower levels of CMD-tryptophan compared to patients without. Statistical analysis was performed using a linear model in generalized estimating equations with amino-acid levels as dependent and depression groups as independent variable. All analyses were adjusted for Hunt & Hess-grade, age, modified-Fisher Score and probe location. DEP_no_ = patients with neither a medical history of depression nor antidepressant medication use 12 months after the subarachnoid hemorrhage; DEP_prior+_ = patients with a medical history of depression; DEP_post+_ = patients with no medical history of depression but with anti-depressant medication use 12 months after the subarachnoid hemorrhage; CMD = cerebral microdialysis; SAH = subarachnoid hemorrhage.

DEP_prior+_ patients showed significantly lower brain tissue levels of CMD-tryptophan (OR = 0.68, CI95 = 0.55–0.83, p < 0.001), CMD-phenylalanine (OR = 0.71, CI95 = 0.58–0.87, p = 0.001) and CMD-tyrosine (OR = 0.7, CI95 = 0.56–0.88, p = 0.002) compared to DEP_no_ patients, adjusted for Hunt & Hess grade, age, modified-Fisher Score and probe location (Fig. 1). No difference was found in the CMD-phenylalanine/tyrosine ratio (p = 0.57).

Associations between preexisting depression and other neuromonitoring parameters are shown in Table 4. There was a trend towards higher CMD-glucose and lower CMD-lactate and CMD-pyruvate levels in DEP_prior+_ patients compared to DEP_no_ patients, which was not statistically significant (p = 0.09, p = 0.11 and p = 0.17, respectively). Markers of systemic inflammation (CRP, leukocyte count) did also not differ between groups (p = 0.8 and p = 0.93, respectively).

**Table 4:** Comparison of neuromonitoring parameters between groups.

Neuromonitoring parameter	DEP_no_ (reference)	DEP_prior+_				DEP_post+_				Deceased			
	Median (IQR)	Median (IQR)	OR	95%CI	p-value	Median (IQR)	OR	95%CI	p-value	Median (IQR)	OR	95%CI	p-value
CMD-Glucose (mmol/l)	1,01 (0,68–1,63)	1,46 (1,05–2,21)	1,16	0,98–1,38	**0,09**	1,29 (0,85–2,05)	1,12	0,93–1,35	0,24	0,94 (0,71–1,31)	0,93	0,81–1,07	0,32
CMD-Lactate (mmol/l)	3,75 (2,61–5,78)	2,93 (2,18–3,39)	0,88	0,74–1,03	0,11	3,23 (2,48–4,20)	0,94	0,82–1,08	0,39	5,75 (3,80–7,44)	1,15	0,98–1,34	**0,08**
CMD-Pyruvate (umol/l)	138,8 (94,2–181,6)	113,4 (80,2–136,5)	0,9	0,77–1,05	0,17	99,9 (74,8–140,9)	0,89	0,78–1,01	**0,07**	123,5 (90,2–153,6)	0,93	0,84–1,04	0,21
CMD-LPR	28,6 (24,2–33,7)	26,4 (23,3–29,9)	0,97	0,90–1,04	0,36	29,7 (24,7–43,2)	1,05	0,93–1,12	0,46	43,8 (34,4–53,0)	1,23	1,10–1,34	0,001*
CMD-Glutamate (umol/l)	13,8 (1,8–92,8)	6,9 (3,2–16,8)	0,84	0,44–1,60	0,6	3,1 (2,2–4,8)	0,72	0,43–1,19	0,2	39,3 (5,2–87,8)	2,25	1,01–5,04	0,05*
Metabolic distress (%)	0 (0–8)	0 (0–7)	0,94	0,84–1,05	0,29	0 (0–72)	1,17	0,87–1,58	0,29	62,5 (14–100)	1,7	1,44–1,99	<0,001*
Mitochondrial dysfunction (%)	10 (0–83)	0 (0–47)	0,91	0,68–1,22	0,53	17 (0–70)	1,01	0,73–1,40	0,96	90 (54–100)	1,42	1,14–1,78	0,002*
Neuroglucopenia (%)	11 (0–33)	0 (0–17)	0,96	0,67–1,38	0,84	0 (0–17)	0,95	0,70–1,28	0,73	23 (6–31)	1,18	0,96–1,45	0,11
ICP crisis (%)	0 (0–4)	0 (0–4)	0,98	0,93–1,03	0,44	0 (0–0)	0,99	0,94–1,04	0,75	0 (0–39)	1,85	1,16–2,97	0,01*
CPP (mmHg)	79,2 (69,4–89,3)	76,1 (69,9–83,8)	1	0,95–1,05	0,88	76,0 (69,3–87,0)	1	0,95–1,05	0,95	83,4 (66,5–79,8)	0,97	0,93–1,01	0,15

Data are shown as n (%) or median (interquartile range). Odds ratios and p-values refer to the comparison of patients with a medical history of depression, patients with antidepressant drug use after 12 months or patients who died to patients with no medical history of depression and no antidepressant drug use after 12 months. An asterisk (*) signifies significant differences, which were only found in patients who died. DEP_no_ = patients with neither a medical history of depression nor antidepressant medication use 12 months after the subarachnoid hemorrhage; DEP_prior+_ = patients with a medical history of depression; DEP_post+_ = patients with no medical history of depression but with anti-depressant medication use 12 months after the subarachnoid hemorrhage; CMD = cerebral microdialysis; IQR = interquartile range; OR = odds ratio; 95% CI = 95% confidence interval; LPR = lactate-to-pyruate-ratio; ICP = intracranial pressure; CPP = cerebral perfusion pressure (mean arterial pressure minus intracranial pressure).

DEP_post+_ patients had significantly lower CMD-tryptophan levels (OR = 0.78, CI95 = 0.63–0.96, p = 0.02) compared to DEP_no_ patients and higher CMD-tryptophan levels than DEP_prior+_ patients (OR = 1.45, CI95 = 1.25–1.68, p < 0.001). We did not observe differences in CMD-phenylalanine (p = 0.2) and CMD-tyrosine concentrations (p = 0.21) between DEP_post+_ patients and DEP_no_ patients, but DEP_post+_ patients had significantly higher CMD-phenylalanine (OR = 1.4, CI95 = 1.18–1.65, p < 0.001) and CMD-tyrosine levels (OR = 1.43, CI95 = 1.2–1.69, p < 0.001) than DEP_prior+_ patients. All analyses were adjusted for Hunt & Hess grade, age, modified Fisher-Score and probe location.

We found a non-significant trend towards lower CMD-pyruvate levels in DEP_post+_ patients compared to DEP_no_ patients (p = 0.07, Table 4). There were no differences in CRP levels and leukocyte count between these groups (p = 0.15 and p = 0.58).

The four patients who died showed similar CMD-tryptophan (p = 0.93), CMD-phenylalanine (p = 0.75) and CMD-tyrosine (p = 0.92) profiles compared to DEP_no_ patients. Regarding other neuromonitoring parameters, patients who died had significantly higher CMD-LPR and CMD-glutamate levels and a more frequent occurrence of metabolic distress, mitochondrial dysfunction and ICP-crises compared to DEP_no_ patients (Table 4).

### Nutritional amino-acid administration and brain interstitial amino-acid levels

The mean daily amounts of nutritional amino-acids were 1243 ± 49 mg tryptophan, 3483 ± 121 mg phenylalanine and 1334 ± 90 mg tyrosine. There was no difference in the amount of amino-acids administered in DEP_no_ patients and DEP_prior+_ patients (tryptophan p = 0.33, phenylalanine p = 0.17, tyrosine p = 0.74). DEP_post+_ patients received more nutritional tyrosine than DEP_no_ patients (1639 ± 140 vs. 1099 ± 110 mg per day, p = 0.035), but a similar amount of tryptophan (p = 0.69) and phenylalanine (p = 0.9). Adjusted for bodyweight, the difference in tyrosine administration was not significant (p = 0.36). In DEP_no_ patients, there was a significant correlation between brain interstitial and nutritional tryptophan and phenylalanine, whereas tyrosine levels correlated in DEP_post+_ patients. No significant correlation was found in DEP_prior +_ patients (Table 5).

**Table 5:** Correlations between cerebral amino-acid levels and amounts of nutritional amino-acids.

	DEP_no_		DEP_prior+_		DEP_post+_	
CMD-tryptophan and nutritional tryptophan	R = 0.26	P = 0.017*	R = 0.09	P = 0.6	R = 0.022	P = 0.86
CMD-phenylalanine and nutritional phenylalanine	R = 0.24	P = 0.027*	R = 0.04	P = 0.81	R = −0.18	P = 0.15
CMD-tyrosine and nutritional tyrosine	R = −0.12	P = 0.27	R = 0.2	P = 0.23	R = 0.62	P < 0.001*

An asterisk (*) signifies significant correlations between the amount of nutritional tryptophan, phenylalanine or tyrosine and respective cerebral amino-acid concentrations per day. Statistical analysis was performed using Spearman’s rho. DEP_no_ = patients with neither a medical history of depression nor antidepressant medication use 12 months after the subarachnoid hemorrhage; DEP_prior+_ = patients with a medical history of depression; DEP_post+_ = patients with no medical history of depression but with anti-depressant medication use 12 months after the subarachnoid hemorrhage; CMD = cerebral microdialysis.

### Functional outcome and brain interstitial amino-acid levels

Three months after SAH, 10 patients (38%) had a good functional outcome. There was no association between functional outcome and CMD-tryptophan (p = 0.47), CMD-phenylalanine (p = 0.29) or CMD-tyrosine (p = 0.36). Of the 16 patients with poor functional outcome who did not die (n = 12), nine (69%) suffered from depression 12 months after SAH. There was an association between depression and poor functional outcome (p = 0.039), which did not remain significant after adjusting for Hunt & Hess grade, gender, age and modified-Fisher score (OR = 8.75, CI95 = 0.85–89.7, p = 0.068).

## Discussion

In this study we found that patients with depressive disorders prior to SAH and patients who took antidepressants 12 months after SAH had lower brain interstitial levels of tryptophan compared to SAH patients without depression, whereas lower levels of CMD-phenylalanine and CMD-tyrosine were only found in patients diagnosed with depression before SAH. Amino-acid levels were not associated with disease severity or hospital complications. In patients without depression we found a significant correlation between nutritional and brain interstitial levels of tryptophan and phenylalanine, which was not present in patients with depression.

Several studies report decreased tryptophan levels in serum of patients with depressive symptoms and disorders^22^. The authors hypothesized that low cerebral tryptophan levels may result in low serotonin levels and therefore lead to the development of depressive disorders. Cerebrospinal fluid (CSF) levels of tryptophan were used as surrogate for brain tissue concentrations in the absence of the possibility of direct parenchymal measurement in humans. Dietary tryptophan depletion resulted in decreased CSF levels of tryptophan^23^, which were in turn associated with depressive relapses in successfully treated patients^24^. However, a poor correlation between blood and CSF tryptophan levels was reported^25^, and, in patients with hepatitis C, interferon alpha treatment was associated with a decrease in peripheral blood tryptophan levels, but did not alter CSF concentrations^26^. This indicates that measurement in blood may not represent the cerebral amino-acid profile. In our study tryptophan levels measured directly in brain tissue were significantly lower in patients with depression, which supports the hypothesis that a lack of serotonin precursor may be involved in the pathophysiology of depressive disorders. The absolute levels in our non-depressed individuals were approximately 4-fold higher than previously reported concentrations of CSF tryptophan and 6-fold lower than plasma levels in healthy controls^27^, which supports the idea of compartmentalization of the central nervous system. However, absolute concentrations should be compared with caution, as they may be influenced by the underlying disease and differing analytical methods. Despite these differences in absolute concentrations, which indicate that blood and CSF levels do not necessarily reflect brain tissue tryptophan levels, the mechanisms underlying decreased concentrations in depressed patients may be similar. Especially chronic inflammation has been discussed as pathophysiologic mechanism underlying diminished tryptophan availability in depressed patients. Pro-inflammatory cytokines induce the enzyme indoleamine 2,3-dioxygenase, which leads to tryptophan catabolism, converting it to kynurenine and potentially neurotoxic and neuroprotective downstream products like quinolinic acid and kynurenic acid^12^. We found no differences in systemic inflammatory parameters between patients with or without depression. This may, however, be strongly influenced by the acute brain injury and infectious complications, potentially shrouding a preexisting condition of chronic inflammation.

Regarding the dopaminergic/noradrenergic pathway, the phenylalanine-tyrosine-ratio was found to be elevated in blood of breast cancer patients suffering from depressive symptoms and interferon alpha treated hepatitis C patients experiencing fatigue^28,29^. This indicates reduced conversion of phenylalanine to tyrosine (and therefore a lack of noradrenalin precursor) in patients with mood disturbances, which may be attributable to a state of chronic inflammation^30^. Pro-inflammatory cytokines induce the formation of reactive oxygen species, which leads to a depletion of tetrahydrobiopterin (BH_4_) pools^31^. BH_4_ is needed as coenzyme for the conversion of phenylalanine into tyrosine and the consecutive conversion of tyrosine into levodopa^11^. In line with this pathophysiologic concept, higher absolute concentrations of phenylalanine and tyrosine were found in plasma of depressed patients compared to healthy controls^32^. In contrast to these previous findings, we measured significantly lower concentrations of both, CMD-phenylalanine and CMD-tyrosine, in the brain tissue of patients with preexisting depression compared to those without. In this context chronic inflammation alone seems not to be a sufficient explanatory model for derangements in the noradrenergic pathway. This may partially explain the failure of trials aiming at alleviating symptoms of depression by BH_4_ administration^33^.

As we found lower CMD levels of all three measured amino-acids in patients with preexisting depression, diminished systemic delivery to the brain may be hypothesized. While we did not measure amino-acid concentrations in blood, we quantified the amount of nutritionally provided amino-acids in our patients and did not find differences between patients with and without depression. Amino-acid levels may also be decreased due to increased consumption. A positron emission tomography study found that patients with major depression showed higher levels of the enzyme monoamine oxidase A, which degrades serotonin and noradrenalin^34^. An increased phenylalanine-tyrosine-ratio and previously reported higher concentrations of phenylalanine and tyrosine in the plasma of depressed patients, however, cannot be explained by this model. The transport of all three amino-acids across the blood-brain-barrier is accomplished by the large neutral amino-acid transporter in a competitive manner, which means that higher concentrations of one amino-acid will increase the flux of this amino-acid across the blood-brain-barrier and suppress the transport of the others^35^. We observed a weak but significant correlation between nutritional and cerebral tryptophan and phenylalanine levels in non-depressed individuals, which was not present in patients with preexisting depression, which may indicate altered transport of amino-acids across the blood-brain-barrier. The strongest correlation was observed between nutritional and brain tissue tyrosine in patients with antidepressant intake after 12 months, which may be attributable to the higher administration of nutritional tyrosine in these individuals (even though it was not significant after adjusting for body weight). The transport of large neutral amino-acids across the blood-brain-barrier is sodium dependent^36^, and therefore relies on a sodium gradient generated by primary active transporters using energy. Altered cerebral energy metabolism and a secondary decrease of transport across the blood brain barrier in depressed patients may be hypothesized. An association of dysregulations in brain energy metabolism and decreased permeability of the blood-brain-barrier with symptoms of melancholia and higher suicidality was described^37^. We did not identify differences in brain energy metabolism assessed by CMD between patients with a medical history of depression, patients with antidepressant intake 12 months after SAH and patients without depression, eventually owing to the acute brain injury determining the metabolic environment.

Furthermore, we observed an increase of all 3 amino acids in the brain extracellular compartment over time, which is consistent with previous findings^14^. This may be explained by enteral and parenteral supplementation in the early phase and improvement of the metabolic state over time which may be accompanied by an enhanced transport of amino acids across the blood-brain-barrier.

Symptoms of depression are common after SAH^3^. The pathophysiologic mechanisms of neuro-psychological disturbances after acute brain injury are incompletely understood. In our cohort patients without a medical history of depression who needed antidepressant medication 12 months after SAH showed decreased levels of brain tryptophan, already in the acute phase after the bleeding. This may indicate a pre-existing alteration in cerebral tryptophan metabolism, suggesting a predisposing metabolic signature for depressive disorders or subclinical depression. There was a trend towards lower phenylalanine and tyrosine concentrations compared to non-depressed patients, which was not statistically significant. Depression was associated with poor functional outcome and poor quality of life after SAH^9^. Tryptophan may be a valuable biomarker for identifying patients at risk for developing depressive disorders after acute brain injury.

The major limitation of this study is that measurements were performed in the brain tissue of patients with SAH, which is known to strongly influence cerebral metabolism. We tested for associations of demographic variables, disease severity and common complications of SAH with amino-acid levels and only found patient age influencing cerebral amino-acid levels. Furthermore, despite amino-acid concentrations being significantly different between the depression groups, there were no alterations in other microdialysis-derived parameters. Patients who died, on the other hand, had a higher lactate-to-pyruvate-ratio, higher CMD-glutamate levels and a more frequent occurrence of metabolic distress, as it is to be expected in patients with poor outcome^38^. These findings indicate that there was no apparent influence of SAH on amino-acid levels and concentrations of tryptophan, phenylalanine and tyrosine seem to be specifically associated with depression. However, this study reveals an association in a particular population sample, but the design does not allow any demonstration of causality. No validation of the HPLC measurement confirming the reported concentrations of amino acids has been performed, however, HPLC is a generally accepted method for the quantification of amino acids and has been utilized earlier for the detection of tryptophan, phenylalanine and tyrosine in human cerebral microdialysate^14,15^. Further limitations include the only local assessment of brain metabolism by CMD. We adjusted all statistical models for the presence of focal brain lesions adjacent to the CMD catheter, however, due to our institutional protocol and current CMD guidelines, probes were almost exclusively located in the frontal lobe. Therefore we cannot exclude different amino-acid concentrations in other brain regions. The assignment to the group of patients with preexisting depression was based on medical history, as a structured interview was not possible in patients with a decreased level of consciousness on admission. Therefore, we cannot provide information on the state of depression our patients were in when the SAH occurred. Similarly, the presence of depression post-SAH was based on the intake of antidepressants at the follow up 12 months after the bleeding. There was no psychiatric follow-up and the indication for antidepressant medication was not documented in our SAH database. Therefore we cannot exclude that patients took antidepressants for other reasons than depression (e.g. anxiety) after SAH. We also cannot exclude that this group or patients classified as having no depression had a lifetime episode of a depressive disorder or suffer from undiagnosed or untreated depression, as the diagnosis of preexisting depression was based on available medical charts/documents. Amino acid administration by enteral and parenteral nutrition may influence cerebral amino acid levels. Importantly, there was no weight-adjusted difference in the amount of administered amino acids between the respective groups. Furthermore, we tried to minimize a potential bias through nutrition and diurnal fluctuations by using CMD samples collected at equivalent points of time.

## Conclusions

Brain tissue concentrations of tryptophan, phenylalanine and tyrosine were significantly lower in patients with a medical history of depression compared to those without. These concentrations were not influenced by disease severity or complications related to SAH. Regarding tryptophan, our results complement previous findings of blood-derived measurements. On the other hand our results contradict an inflammation-related inhibition of phenylalanine and tyrosine processing and argue for alternative pathophysiologic models, such as an increased cerebral demand or altered transport across the blood-brain-barrier.

## Data Availability

The datasets generated during and/or analyzed during the current study are available from the corresponding author on reasonable request.
